# Gene Expression Profile of Stromal Factors in Cancer-Associated Fibroblasts from Prostate Cancer

**DOI:** 10.3390/diagnostics12071605

**Published:** 2022-06-30

**Authors:** Noemi Eiro, Jesús María Fernández-Gómez, Cristina Gonzalez-Ruiz de León, Maria Fraile, Jorge Gonzalez-Suarez, Beatriz Lobo-Rodríguez, Jorge García-Rodríguez, Safwan Escaf, Francisco J. Vizoso

**Affiliations:** 1 Research Unit, Fundación Hospital de Jove, Avda. Eduardo Castro 161, 33920 Gijón, Spain; maria.fraile82@gmail.com; 2 Department of Urology, Hospital Universitario Central de Asturias, Universidad de Oviedo, 33011 Oviedo, Spain; jmfergomez@gmail.com (J.M.F.-G.); cgruizleon@gmail.com (C.G.-R.d.L.); jorgemangonzalez@gmail.com (J.G.-S.); her2000@hotmail.es (B.L.-R.); jgrmed@hotmail.com (J.G.-R.); escafsafwan@gmail.com (S.E.)

**Keywords:** prostate cancer, cancer-associated fibroblasts CAFs, tumor stroma, metastatic cancer, androgen deprivation therapy, MMP11

## Abstract

Recent investigations point at the stromal microenvironment to assess additional diagnostic information and provide new therapeutic targets in cancer. The aim of the study was to contribute to the characterization of the phenotype of cancer-associated fibroblasts (CAFs) in prostate cancer (PCa) compared with normal prostate-associated fibroblasts (NAFs) and fibroblasts from benign prostatic hyperplasia (BPH). Three patient populations were prospectively recruited: 23 patients with new localized PCa, 14 patients with advanced PCa treated with androgenic deprivation therapy (ADT), and 7 patients with BPH. Gene expression of 20 stroma-derived factors, including the androgen receptor (AR), chaperones (HSPA1A and HSF1), growth factors (FGF2, FGF7, FGF10, HGF, PDGFB, and TGFβ), proteins implicated in invasion (MMP2, MMP9, and MMP11), inflammation (IL6, IL17RB, NFκB, and STAT3), and in-stroma/epithelium interaction (CDH11, CXCL12, CXCL14, and FAP), was evaluated. Localized PCa CAFs showed a significant higher expression of FGF7, IL6, MMP2, and MMP11 compared with NAFs or IL17RB compared with BPH fibroblasts, but significantly lower expression of FGF10 and IL17RB compared with NAFs or CXCL14 compared with BPH fibroblasts. In addition, CAFs from ADT-resistant PCa showed significantly higher MMP11 and NFκB but significant lower TGFβ expression compared with CAFs from ADT-sensitive tumors. Our results contribute to defining the CAFs phenotypes associated to PCa progression, which may contribute to the diagnosis and design of alternative therapies in PCa.

## 1. Introduction

Prostate cancer (PCa) is the most common male cancer in western societies. Between 1% and 33% of surgical-treated PCa will eventually become metastatic [1,2]. Although epithelial differentiation, noted by the Gleason score at the time of diagnosis, helps with prognosis and management, it is not sufficient for a more accurate prediction of metastasis development. Recent investigations point at the stromal microenvironment to assess additional diagnostic information and provide new therapeutic targets in cancer.

Prostate development and homeostasis require bidirectional signaling between epithelial cells and stromal constituents, which show a stromal-to-epithelial ratio of 2:1. Fibroblasts are the most important stromal cell type. They are tasked with synthesizing and depositing extracellular matrix components, allowing other cells to settle and migrate along a three-dimensional support and thereby generate an organ-specific architecture. In addition, these are very versatile cells producing bioactive molecules, which are involved in several physiological processes, such as angiogenesis and tissue repair [3]. However, the normal two-way signaling between epithelial cells and stromal constituents is disrupted in cancer, where the stroma becomes disorganized. In this pathological situation, normal prostatic fibroblasts are replaced by activated cancer-associated fibroblasts (CAFs), which are the major microenvironment components of the prostate tumor. Compared with normal fibroblasts, CAFs exhibit increased proliferation and migratory behavior. Although the mechanisms involved remain misunderstood, it is known that CAFs contribute to creating a favorable microenvironment for cancer cells by releasing high levels of growth factors, cytokines, chemokines, metalloproteases (MMPs), and exosomes [4]. Across all of these stromal factors, CAFs alter the deposition of the extracellular matrix and induce malignancy in non-tumorigenic prostate epithelial cells [5]. In addition, CAFs could mediate inflammation and angiogenesis by recruiting macrophages to stimulate angiogenesis, which may then promote tumor growth [6].

Genomic-level studies have identified CAFs-specific prognosis gene signatures in PCa [7,8]. In this sense, we have recently shown CAFs heterogeneity among PCa in terms of certain molecular profile expressions that may be relevant in tumor development, progression, and androgen deprivation therapy (ADT) resistance [9].

The aim of the present study was to contribute to the characterization of the CAFs phenotype in PCa in comparison to normal prostate-associated fibroblasts (NAFs) and BPH fibroblasts. In order to carry this out, the gene expression of 20 stroma-derived factors, including the androgen receptor (AR), chaperones (HSPA1A and HSF1), growth factors (FGF2, FGF7, FGF10, HGF, PDGFB, and TGFβ), protein implicated in invasion (MMP2, MMP9, and MMP11), inflammation (IL6, IL17RB, NFκB, and STAT3), and in-stroma/epithelium interaction (CDH11, CXCL12, CXCL14, and FAP), was evaluated in cultured fibroblast-like cells obtained by transrectal ultrasound biopsy from patients with new diagnosis of localized PCa or advanced PCa treated with ADT and from patients with BPH.

## 2. Material and Methods

### 2.1. Patients

The following patient populations, enrolled prospectively from 2012 to 2018, were included in the present study:

- Seven patients with BPH underwent prostate TUR (transurethral resection; mean age: 72 years; sd: 9.38), where TUR samples were collected.
- Twenty-three patients with suspected PCa who underwent radical prostatectomy (mean age: 66.96 years; sd: 6.57). These patients were selected with an estimated survival of more than 10 years since the tumor was clinically localized (cT2 or less; PSA < 20 nanograms per milliliter (ng/mL). Patients with estimated survival under 10 years, with significant local involvement by rectal examination or transrectal ultrasound, with PSA > 20 ng/mL and/or suspected lymph node or metastasis in imaging studies (CT and bone scan), with previous neoplasms, with neoadjuvant therapy, with development of a second primary tumor, or with an insufficient amount of tissue obtained for culture cells were excluded. In the eventually selected patients, open radical or laparoscopic prostatectomy was performed using standardized techniques. Pelvic lymphadenectomy was performed when the PSA was above 10 ng/mL and/or a Gleason score greater than 6 was found in the biopsy sample. Once the radical prostatectomy was performed, the stage and the Gleason pattern of the specimen, the presence of perineural or seminal vesicles invasion, and the occurrence of positive margins or pathological lymph node involvement (when lymphadenectomy was performed) were analyzed.
- Fourteen advanced PCa patients already diagnosed and treated with androgen deprivation therapy (ADT; mean age: 76.71 years; sd: 8.07). A new transrectal ultrasound biopsy for prostate carcinoma was performed as described below. ADT-sensitive prostate tumors were defined as having indetectable PSA and tumor disappearance upon image-based diagnosis. ADT-resistant prostate tumors were selected based on the European Association of Urology (EAU) criteria: serum testosterone castration levels (testosterone < 50 ng/dL) and three consecutive rises of PSA 1 week apart, resulting in two 50% increases over the nadir, with a PSA > 2 ng/mL or appearance of two or more lesions on bone scan or soft tissue lesions. The median follow-up for patients with ADT-sensible tumors was 38 months (range of 2–93) and 21 months (range of 20–30) for patients with ADT-resistant tumors.

The clinical–pathological characteristics of patients whose prostatectomy specimens were analyzed, including age, tumor stage (T), Gleason score, and PSA of the biopsy specimen, are shown in Table 1. The pathological stage (pT) and the Gleason score of the localized tumors corresponded to those of the radical prostatectomy specimen. The local stage of the tumors treated with ADT (castration-sensitive or castration-resistant) is not shown in the table. In these cases, the Gleason score of the biopsy is included since radical prostatectomy was not performed.

**Figure 1:**
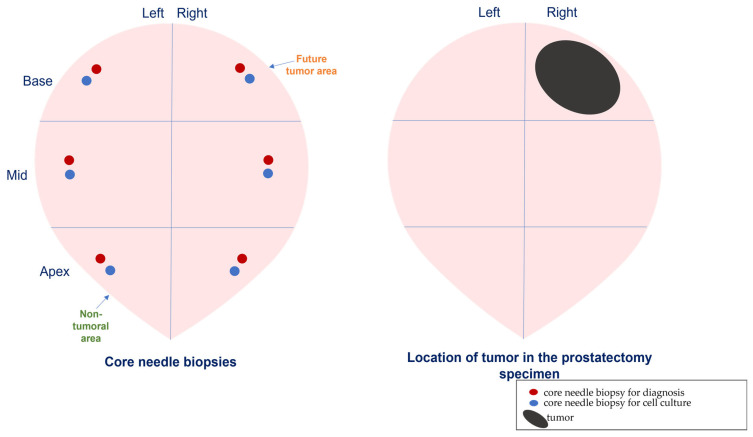
Schematic representation of cores’ biopsy obtention and selection.

Prostate biopsy samples were taken from all PCa patients using transrectal ultrasonography (TRUS). Generally, except for large prostates (Vienna nomograms), 12-core needle biopsies were obtained from the peripheral region using local anesthesia. Two-core needle biopsies were taken from each area (base, mid, and apex, from left and right side): one for diagnosis and one for cell culture (Figure 1). All 6-core needle biopsies were cultured but for the molecular study, we selected cores according to the tumor location in the radical prostatectomy specimen. Therefore, CAFs are fibroblasts isolated from the tumor area and NAFs are fibroblasts from distant non-tumoral areas. In multifocal tumors, two different tumor areas were analyzed, considering the main tumor area the one with the highest grade or stage.

The project was approved by the Regional Clinical Research Ethics Committee (reference 09/2012). Patients received detailed information about the nature and purpose of the research. Specific written consent was required for all patients.

### 2.2. Isolation and Culture of Fibroblasts from Surgical or Biopsy Specimens

As described previously [9], biopsy specimens were seeded in a 12-well plate and incubated with type I collagenase (1.25 mg/mL) in Dulbecco’s Modified Eagle Medium: Nutrient Mixture F-12 (DMEM/F-12) medium with 10% Fetal Bovine Serum (FBS) for 48 h at 37 °C in a humidified atmosphere of 5% CO_2_ and 95% air. Then, at the semi-confluence, the fibroblasts were selected by differential trypsinization and after various passages, only fibroblasts, characterized by their spindle-shape morphology, were visible.

The purity of the cultured fibroblasts was confirmed by the characterization of positive vimentin and alpha-actin, and the negative desmin by immunohistochemistry and by flow cytometry with the antibody CD90 clone AS02.

### 2.3. qRT-PCR

The RNeasy Mini Kit (Qiagen, Hilden, Germany) was used for full RNA isolation according to the manufacturer’s instructions. For the cDNA synthesis, the Transcriptor First Strand cDNA Synthesis Kit (Roche, Mannheim, Germany) was used as previously described [9]. Expression levels of genes were evaluated by real-time PCR using RealTime-ready custom panel plates (Roche, Mannheim, Germany) on a LightCycler 480 II (Roche, Mannheim, Germany). The custom-designed plates contained primers and probes specific for the factors analyzed (Table 2). All 44 patients were included in the RT-PCR analysis performed in triplicate. Data represent mean ± SD.

### 2.4. Immunostaining Analyses

Immunostaining analyses were performed to evaluate the expression of FGF7 (Abcam, Cambridge, United Kingdom, Ab90259, 1 µg/mL, 15 min), IL6 (AbNova, Taipéi, Taiwán, H0003569-B01P, 1/300, 2 h), MMP2 (ThermoFisher Scientific, Waltham, Massachusetts, USA, MS806, 1/100, 2 h) and MMP11 (ThermoFisher Scientific, Waltham, Massachusetts, USA, MA5-32285, 1/500, 2 h) in NAFs, CAFs, and MMP11 in both cultured CAFs and in prostatectomy tissue specimens. Cultured CAFs were fixed by adding ice-cold methanol for 20 min. Tissue samples were routinely fixed in 10% neutral-buffered formalin and stored after being embedded in paraffin at room temperature in archives of the Department of Anatomy and Pathology. Serial 5 μm sections were consecutively cut with a microtome (Leica Microsystems GmbH, Wetzlar, Germany) and transferred to adhesive-coated slides. To enhance antigen retrieval, slides were treated in a PT-Link^®^ (Dako, Glostrup, Denmark) at 97 °C for 20 min; in citrate buffer, pH 6.1, for FGF7 and IL-6; or in Tris-EDTA buffer, pH 9, for MMP2 and then washed in phosphate-buffered saline (PBS). The immunostaining was carried out using a TechMate TM50 autostainer (Dako, Glostrup, Denmark). Endogenous peroxidase activity was blocked by incubating the slides in peroxidase-blocking solution (Dako, Glostrup, Denmark) for 5 min. The EnVision Detection Kit (Dako, Glostrup, Denmark) was used as the staining detection system. Sections were counterstained with hematoxylin, dehydrated with ethanol, and permanently cover-slipped.

### 2.5. Statistical Analyses

Clinical and pathological features of the patients (age, clinical stage, and Gleason score) were analyzed to study their relationship with stromal characteristics in PCa. Parametric analyses were performed, including Student’s *t*-test and one-way ANOVA for variables with normal distribution. Conversely, non-parametric tests (Mann–Whitney and Kruskal–Wallis) were used when the Kolomogrov–Smirnov test indicated a non-normal data distribution. Only significant differences in the factor comparation are shown. A *p*-value of less than 0.05 was considered statistically significant. PASW Statistics 18 (Chicago, IL, USA) was used to perform the statistical analyses.

## 3. Results

### 3.1. Molecular Profile of CAFs and Paired NAFs

**Figure 2:**
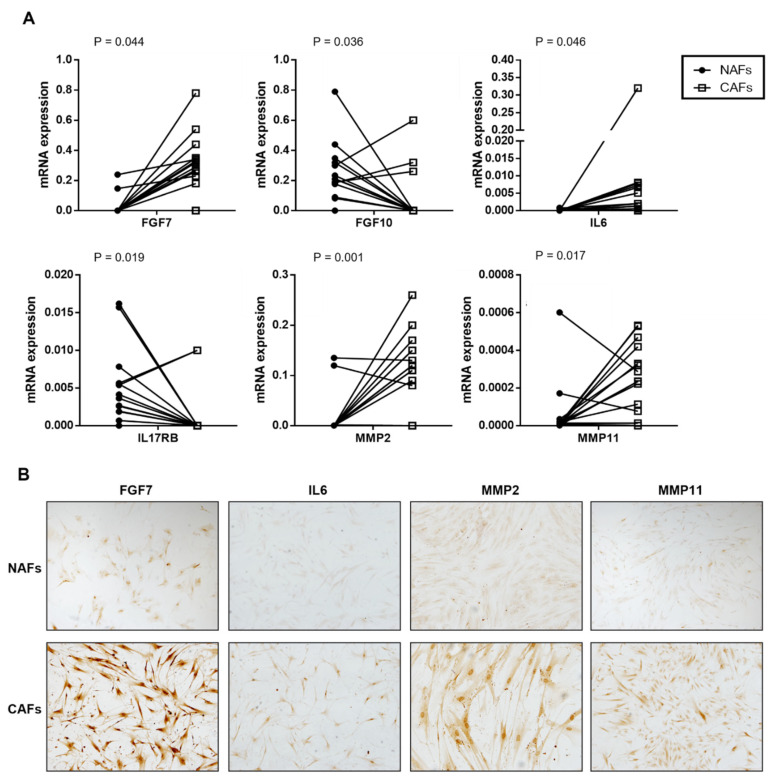
(**A**) Gene expression of stromal factors by CAFs and paired NAFs from 15 prostate carcinomas. (**B**) FGF7, IL6, MMP2, and MMP11 protein expression of cultured NAFs (upper panel) and CAFs (lower panel).

Of the 20 factors analyzed by qRT- PCR, we found significant differences in the expression of several factors between CAFs and NAFs in 15 cases, in which both paired samples were analyzed. CAFs showed a higher expression of FGF7, IL6, MMP2, and MMP11 compared with paired NAFs, while NAFs showed a significantly higher expression of FGF10 and IL17RB (Figure 2A). We have evaluated the protein expression of factors upregulated in CAFs (FGF7, IL6, MMP2, and MMP11). As it shown in Figure 2B, CAFs showed a higher expression compared with NAFs.

### 3.2. Comparing the Gene Expression Profile of CAFs from Multifocal Tumors

**Figure 3:**
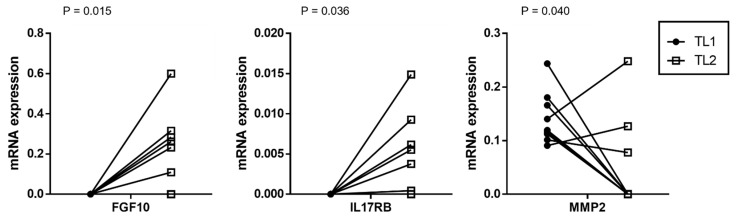
Gene expression of stromal factors by CAFs from two foci of nine multifocal prostate carcinomas: main tumor location (TL1) and secondary tumor focus (TL2).

A total of nine cases of the twenty-three localized PCa had multifocal tumors; the main focus is the one with the highest grade or stage (Tumor Location (TL1)) and a secondary focus was used (TL2). When comparing the gene profile of CAFs from two tumor foci for each one of these cases, we only found significant differences between CAFs populations for FGF10, IL17RB, and MMP2 (Figure 3), evidencing a little variability within the multifocal PCa tumor focus.

### 3.3. Comparing the Gene Expression Profile of NAFs or CAFs with Fibroblasts from BPH

**Figure 4:**
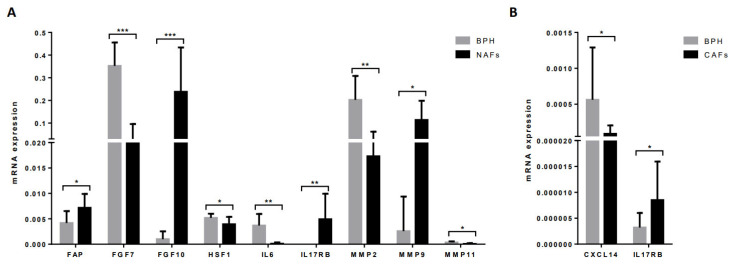
Gene expression of stromal factors by (**A**) NAFs or (**B**) CAFs from prostate tumors compared with fibroblasts from benign prostate hyperplasia (BPH). * *p* ≤ 0.05; ** *p* < 0.01; *** *p* < 0.001.

NAFs showed significantly higher expressions of FAP, FGF10, IL17RB, and MMP9 but significantly lower expressions of FGF7, HSF1, IL6, MMP2, and MMP11 compared with BPH fibroblasts (Figure 4A).

CAFs showed a significantly higher expression of IL17RB but a significant lower CXCL14 expression compared with BPH fibroblasts (Figure 4B).

### 3.4. Gene Expression Profile of CAFs According to Clinical–Pathological Characteristics of Patients and Tumors

**Figure 5:**
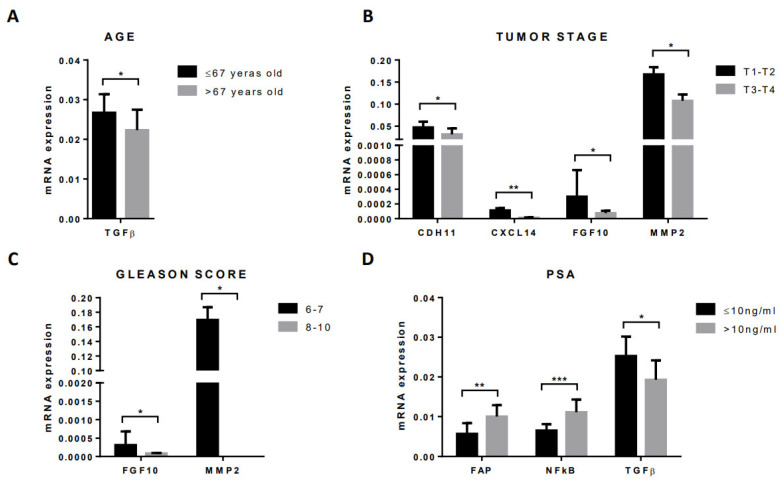
Gene expression of stromal factors by CAFs according to clinical–pathological characteristics of prostate carcinomas, (**A**) age, (**B**) tumor stage, (**C**) Gleason score, and (**D**) PSA level. * *p* ≤ 0.05; ** *p* < 0.01; *** *p* < 0.001.

The analysis of the relationship between stromal factors’ expression by CAFs and clinical–pathological characteristics in patients with localized tumors from patients who underwent radical prostatectomy shows significant associations with patient age, serum levels of pre-treatment PSA, tumor stage, and Gleason score (Figure 5). Younger patients (age ≤67 years) had higher TGFβ gene expression than older patients (age >67 years; Figure 5A). Regarding the pathological stage, either CAFs from T1–T2 tumors showed a higher expression of CDH11, CXCL14, FGF10, and MMP2 than those ones from T3–T4 tumors (Figure 5B). In addition, tumors with a low Gleason score (6–7) showed a higher gene expression of FGF10 and MMP2 than tumors with a high Gleason score (8–10; Figure 5C). Pre-treated PSA serum levels correlated positively with FAP and NFκB gene expression by CAFs but correlated negatively with TGFβ gene expression (Figure 5D).

### 3.5. Gene Expression Profile of CAFs from Tumors of Patients Undergoing Androgen Deprivation Therapy

In the present study, we also analyzed the gene expression of CAFs from 14 patients with previously diagnosed patients with advanced PCa and receiving ADT.

**Figure 6:**
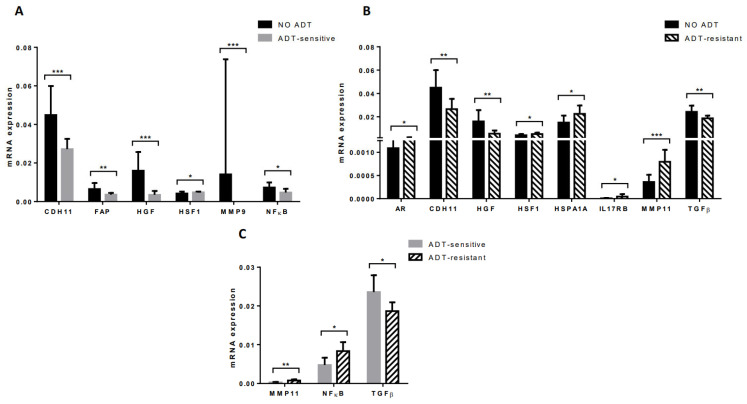
Gene expression of stromal factors by CAFs from prostate carcinoma of patients under androgen deprivation therapy. No-ADT compared with (**A**) ADT-sensitive and (**B**) ADT-resistant, and (**C**) ADT-sensitive compared with ADT-resistant. * *p* ≤ 0.05; ** *p* < 0.01; *** *p* < 0.001.

CAFs from ADT-sensitive tumors (*n* = 7) had a significant lower expression of CDH11, FAP, HGF, MMP9, and NFκB, but a higher expression of HSF1 compared with CAFs from localized ADT-treated tumors (Figure 6A). CAFs from ADT-resistant tumors (*n* = 7) had a significant higher expression of AR, HSF1, HSPA1A, IL17RB, and MMP11, but a lower expression of CDH11, HGF, and TGFβ compared with CAFs from localized ADT-treated tumors (Figure 6B). Therefore, these findings led us to consider that ADT may induce changes in the CAFs gene expression profile.

On the other hand, CAFs from ADT-sensitive tumors showed a high TGFβ gene expression whereas CAFs from ADT-resistant tumors showed a higher NFκB and MMP11 expression (Figure 6C), suggesting molecular changes induced by the ADT response.

Aiming to validate the gene expression, we evaluated the protein expression of MMP11 due to its known role in cancer progression [9] and its possible role in predicting ADT response in both cultured CAFs and cancer tissues from radical prostatectomy. As shown in Supplementary Figure S1, when cultured CAFs obtained from biopsy exhibited a high MMP11 gene expression, both cultured CAFs and CAFs from the corresponding prostatectomy cancer tissue also showed a high protein expression; the same occurs with a low gene expression (Supplementary Figure S1).

## 4. Discussion

**Figure 7:**
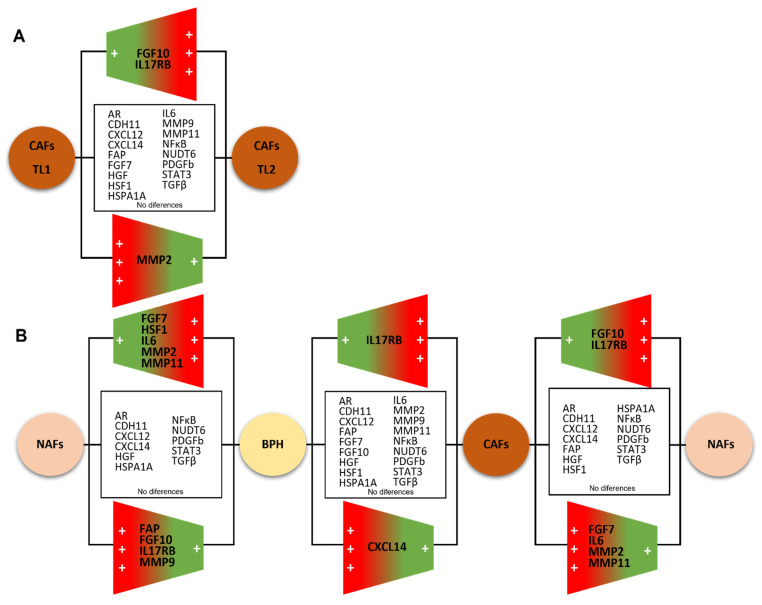
Scheme summarizing the comparative analysis of gene expression of stromal factors among (**A**) CAFs from main tumor location (TL1) and secondary tumor focus (TL2); (**B**) NAFs and CAFs from prostate carcinomas and fibroblasts from benign prostatic hyperplasia (BPH). The upregulation (red), downregulation (green), or non-changed factors for each factor in each comparison are shown.

There are studies indicating that CAFs can originate from resident fibroblasts, adipocytes, bone marrow-derived mesenchymal cells and epithelial cells, or endothelial cells through the epithelial or endothelial-to-mesenchymal transition [10]. Even so, although the prostate gland is a confined organ, our results show significant differences in the gene expression of stromal factors between cultured CAFs and paired NAFs from PCa, which seems to correspond to local stromal changes associated with this neoplasia. In addition, fibroblasts from BPH exhibit significant differences compared with NAFs or CAFs. Thus, these findings seem to indicate that cultured fibroblasts maintain their differentiated patterns’ expression, at least for some time after separation from either benign epithelial or cancer cells. On the other hand, although we accounted for a small number of cases of multifocal tumors in our prospective series of patients, our findings indicate that CAFs from the secondary tumor focus have a similar phenotype compared with CAFs from the main focus, at least for the majority of the analyzed factors in the present study (Figure 7A). Although an intra-tumor and inter-tumor genomic heterogeneity of multifocal localized PCa [11,12] has been reported, it is possible that CAFs show less genetic alterations, which might make it interesting to consider CAFs as an anti-tumor therapeutic target.

Figure 7B represents a scheme summarizing the comparative gene expression analysis of CAFs and paired NAFs in our study population of patients with PCa as well as with fibroblasts from BPH specimens. CAFs showed a higher expression of FGF7, IL6, MMP2, and MMP11, while also showing a lower expression of FGF10 and IL17RB than NAFs. Interestingly, we previously demonstrated that CAFs from breast carcinomas also show a significantly higher expression of these same factors by CAFs than their corresponding paired NAFs [13], influencing cancer cell behavior [14]. Thus, it seems that these stromal factors may have a key role in tumor development in both malignancies. These findings on stromal biology appear to support the hypothesis of the similarities in the biological, genetic, and epidemiological aspects of breast and prostate cancer, which dates back to the 1950s [15]. Taken together, our results point to the high gene expressions of FGF7, IL6, MMP2, and MMP11, defining a pro-tumoral molecular profile of CAFs in PCa. In fact, there are several datum pointing to a role of these stromal factors in tumor progression. IL6 increases proliferation and protects PCa cells against apoptosis [16]. IL6 is also associated with the neuroendocrine differentiation of PCa cells, which is a recognized factor of PCa aggressiveness. In addition, the elevation of serum levels of IL6 or activation of IL6-signaling pathways in the tumor tissue correlates significantly with the shortened overall survival and time to progression in PCa [17].

Our results show an inverse pattern of expression of FGF7 and FGF10 between NAFs and CAFs, with a higher FGF10 expression by NAFs. At present, we have no reasonable explanation for these differences between NAFs and CAFs, which we did not find in breast cancer [13]. Fibroblast growth factors (FGFs) have many important functions during the embryonic period as well as many diseases. They perform specific directional signaling from the stroma to the epithelium, contributing to the homeostasis between the two compartments. FGF7 and FGF10 are only expressed by stromal cells in the prostate and they constitute the signal part of androgen-modulated directionally specific communication from stroma to epithelial cells via resident epithelial cell FGFR2IIIb. Interestingly, the existence of two distinct subtypes of stromal cells in prostate tumors was reported: one exhibited a fibroblast-like morphology and did not display smooth muscle cell (SMC) α-actin, whereas the other exhibited SMC α-actin and an SMC-like morphology in vitro, for instance, as shown by the CAFs in our study. Both subtypes expressed FGF7, whereas the absence of FGF10 was observed in the latter cell stromal subtype. Considering that FGF10 is found expressed at an extremely low amount in normal adult prostate tissue [9], these results demonstrate stromal cell heterogeneity in the signal reception of FGFs from the epithelium, which may be a determinant of whether tumors remain in non-malignant homeostasis or progress to malignancy.

MMPs degrading the stromal connective tissue and basal membrane components are key elements of tumor invasion and metastasis. In general, MMPs were associated with tumor aggressiveness and/or poor prognostic in patients with PCa [18]. Accordingly, we found greater expression of MMP2 and MMP11 by CAFs than by paired NAFs in PCa. In particular, MMP11 (also known as stromelysin-3) expression by the intratumor stroma has been reportedly correlated with the aggressive phenotype and poor clinical outcome in several solid tumors, such as breast cancer [19,20,21], where its expression seems to be associated with an inflammatory phenotype [22,23], and also in PCa [24,25]. Although, compared with other MMPs, MMP11 has relatively low proteolytic potential and there are other mechanisms which could explain the special relevance of MMP11 in tumor progression. Thus, while the majority of MMPs are secreted as proenzymes that require extracellular activation, MMP11 is processed intracellularly and secreted as an active enzyme. This also suggests that this endopeptidase may have a unique role in tumor growth and progression [26,27]. It has been revealed, as well, that tumorigenesis induced by MMP11 does not result from increased cancer cell proliferation but from decreased cancer cell death through apoptosis and necrosis, indicating that the cellular function of MMP11 is in favor of cancer cell survival in the stromal environment [28]. On the other hand, there is evidence that MMP11 may alter the stromal microenvironment of human carcinomas to stimulate tumor angiogenesis [29].

In the present study, we also compared stromal factor gene expressions between NAFs or CAFs and fibroblasts from BPH specimens. BPH is a non-malignant growth of the prostate typically occurring in older men, which consists of an alteration of prostate tissue homeostasis from the central zone, while prostate cancer usually develops in the peripheral zone. Expressions of FGF7, IL6, MMP2, and MMP11 were higher in BPH fibroblasts compared with NAFs and in NAFs compared with paired CAFs. This might indicate the implication of these factors in the deregulation of the epithelial–stromal interaction responsible for the initiation and/or promotion of these proliferative diseases of the aging human prostate [30]. However, NAFs from patients with PCa showed a higher expression of FAP, FGF10, IL17RB, and MMP9 compared with fibroblasts from BPH, which might correspond to the possible molecular influences of the presence of tumors in the organ confined to a gland. In this context and with regard to FAP, it is interesting to say that this factor is a cell-surface bound protease, for which increased expression was associated with the most aggressive “matrix remodeling” [10].

Regarding clinical–pathological characteristics from patients with localized PCa who underwent radical prostatectomy, our results showed some significant associations of the CAFs molecular profile with age, pre-treatment PSA serum levels, pathologic stage, and Gleason score, which also suggests the involvement of CAFs in tumor biology. Our finding of a positive association between pre-treatment PSA serum levels, which is a recognized factor of poor prognosis in PCa [31], and both NFκB and FAP gene expressions by CAFs is worthy to note. It has been reported that NF-κB promotes cell survival, tumor invasion, metastasis, and chemoresistance in PCa [32].

Androgen receptors (AR)-regulated pathways are critical for PCa cell proliferation and survival. Therefore, AR-targeting therapies are a mainstay of prostate cancer treatment based primarily on the observed effects on cell growth, resistance to treatment, and metastasis in malignant epithelial cells [33]. Nevertheless, AR is also instrumental in the development and maintenance of non-malignant cells in the prostate stroma. In this sense, our data suggest that ADT induced a lower gene expression of CDH11, FAP, HGF, MMP9, and NFκB in CAFs from ADT-sensitive tumors compared with non-treated tumors. These findings are in accordance with evidence suggesting that ADT-induced stromal remodeling is accompanied by a functional transformation of the prostatic stromal environment [34]. In this line, previous studies have shown that CAFs express functional AR and play a role in the development and progression of prostate cancer. Although the AR roles in CAFs are still unclear, it has been shown that prostate cancer epithelial growth, invasion, and colony formation abilities decreased when knocking down the CAFs AR. In addition, an earlier study found that the IGF1, FGF7, FGF10, SDF1, HGF, and TGFβ2 expression levels decreased in the AR, which was knocked down to CAFs [35]. However, in the present study, when we compared non-treated tumors with ADT-resistant tumors, an increased expression of HSPA1A, IL17RB, and, specially, MMP11 by CAFs was observed in these. In addition, we found that CAFs from these ADT-resistant tumors had a high MMP11 expression compared with CAFs from ADT-sensitive tumors, as we reported in a previous study [9]. Thus, these results appear to agree with some experimental observations that CAFs expressing pro-tumor factors could have a role in the development of ADT resistance [36]. In this context, the consideration of MMP11 gene expression by CAFs as a prognostic and predictive factor may help address the lack of prognostic tools in PCa that can identify patients who are at risk for lethal metastasis.

The heterogeneity of CAFs in terms of their origin and phenotype has an impact on distinct functions, such as tumor growth, stromal remodeling, the angiogenic process, drug resistance, and metastasis development. In this sense, therapeutic targets according to the CAFs molecular profile have been proposed [37]. However, as Padrip De et al. stated, “the state-of-art management of today’s disease does not necessarily include a CAF-inclusive therapy. We are just beginning to appreciate that the knowledge about the CAF functions and inhibition is critical in managing the disease towards developing a CAF-inclusive therapy” [38].

One of the limitations of the current prospective study is that the follow-up period may not have been long enough in our patients. It is known that more than 90% of biochemical recurrences occur within 5 years after radical prostatectomy [1]. Other limitations are the lack of a comparison of the gene expression of cultured fibroblasts and laser-captured fibroblasts from bothc the same patient and the number of cases in each group studied.

In summary, our results contribute to defining the CAFs molecular profile associated with PCa. In addition, our data suggest that stromal fibroblasts’ gene expression is not just a reaction to the presence of the tumor but CAFs seem to be active components in the tumor development. These findings may add to the diagnosis and design of new strategies based on the tumor stroma as a therapeutic target in PCa. The results of this study help to characterize and therefore differentiate the phenotypes of fibroblasts, for example, from a benign condition such as BPH and from a non-tumor and tumor area. Additionally, MMP11 gene expression by CAFs may help in the prognostic and predictive response to ADT. Furthermore, this last aspect may be a special opportunity if we consider that PCa stromal cells do not usually harbor somatic genomic mutations [39].

## Figures and Tables

**Table 1:** Basal characteristics of patients with prostate carcinoma.

Characteristics	Localized PCa (*n* = 23)	PCa ADT-Sensitive (*n* = 7)	PCa ADT-Resistant (*n* = 7)
	N (%)	N (%)	N (%)
**Mean Age (years)**	66.9 ± 1.3	78.8 ± 1.9	74.5 ± 3.8
Tumor Stage (T) *			
T1-T2	17 (73.9)	-	-
T3-T4	4 (17.4)	-	-
Gleason Score *			
6-7	16 (69.6)	1 (14.3)	1 (14.3)
8-10	5 (21.7)	6 (85.7)	6 (85.7)
**PSA (ng/mL)**			
≤10	19 (82.6)	4 (57.1)	1 (14.3)
>10	4 (17.4)	3 (42.9)	6 (85.7)

Tumor stage (T) *: 2 cases of localized PCa were lost. Gleason score *: Localized PCa from radical prostatectomy and PCa castration-sensitive and resistant from biopsy.

**Table 2:** Stromal factor analyzed in cultured fibroblasts from patients with prostate cancer and BPH.

Factor	Gene Name	Reference
**Hormone Receptors**		
AR	Androgen Receptor	102544
**Proteins Implicated in Stroma/Epithelium Interactions**		
CDH11	Cadherin-11	112371
CXCL12	Stromal cell derived factor 1	110618
CXCL14	Chemokine ligand 14	111260
FAP	Fibroblast activation protein alpha	108274
**Growth Factors**		
FGF7	Fibroblast growth factor 7	113109
FGF10	Fibroblast growth factor 10	118274
HGF	Hepatocyte growth factor	108357
FGF2	Fibroblast growth factor 2 (NUTD6)	118274
PDGFB	Platelet-derived growth factor beta	110713
TGFβ	Transforming growth factor beta	101210
	**Chaperones**	
HSPA1A	Heat Shock Protein Family A (Hsp70) Member 1A	145875
HSF1	Heat shock transcription factor 1	110674
**Proteins Implicated in Inflammation**		
IL6	Interleukin 6	113614
IL17RB	Receptor of interleukine 17	140392
NFkB	Nuclear factor kappa B	100646
STAT3	Signal transducer and activation of transcripcion 3	110694
**Proteins Implicated in Invasion**		
MMP2	Matrix metalloprotease 2	103899
MMP9	Matrix metalloprotease 9	139820
MMP11	Matrix metalloprotease 11	103163

## Supplementary Materials

The following supporting information can be downloaded at: www.mdpi.com/article/10.3390/diagnostics12071605/s1, Figure S1: Representative examples of high and low MMP11 expression, both qRT-PCR transcript level (A) and protein (B) by CAFs (upper panel) and by prostatectomy cancer tissue (lower panel).

## References

[1] Amling C.L., Blute M.L., Bergstralh E.J., Seay T.M., Slezak J., Zincke H. (2000). Long-term hazard of progression after radical prostatectomy for clinically localized prostate cancer: Continued risk of biochemical failure after 5 years. J. Urol..

[2] Freedland S.J., Humphreys E.B., Mangold L.A., Eisenberger M., Dorey F.J., Walsh P.C., Partin A.W. (2005). Risk of prostate cancer-specific mortality following biochemical recurrence after radical prostatectomy. JAMA.

[3] Costa-Almeida R., Soares R., Granja P.L. (2018). Fibroblasts as maestros orchestrating tissue regeneration. J. Tissue Eng. Regen. Med..

[4] Bonollo F., Thalmann G.N., Kruithof-de Julio M., Karkampouna S. (2020). The Role of Cancer-Associated Fibroblasts in Prostate Cancer Tumorigenesis. Cancers.

[5] Bhowmick N.A., Neilson E.G., Moses H.L. (2004). Stromal fibroblasts in cancer initiation and progression. Nature.

[6] Erez N., Truitt M., Olson P., Arron S.T., Hanahan D. (2010). Cancer-Associated Fibroblasts Are Activated in Incipient Neoplasia to Orchestrate Tumor-Promoting Inflammation in an NF-kappaB-Dependent Manner. Cancer Cell.

[7] Planche A., Bacac M., Provero P., Fusco C., Delorenzi M., Stehle J.C., Stamenkovic I. (2011). Identification of prognostic molecular features in the reactive stroma of human breast and prostate cancer. PLoS ONE.

[8] Wikstrom P., Marusic J., Stattin P., Bergh A. (2009). Low stroma androgen receptor level in normal and tumor prostate tissue is related to poor outcome in prostate cancer patients. Prostate.

[9] Eiro N., Fernandez-Gomez J., Sacristan R., Fernandez-Garcia B., Lobo B., Gonzalez-Suarez J., Quintas A., Escaf S., Vizoso F.J. (2017). Stromal factors involved in human prostate cancer development, progression and castration resistance. J. Cancer Res. Clin. Oncol..

[10] Bussard K.M., Mutkus L., Stumpf K., Gomez-Manzano C., Marini F.C. (2016). Tumor-associated stromal cells as key contributors to the tumor microenvironment. Breast Cancer Res..

[11] Lovf M., Zhao S., Axcrona U., Johannessen B., Bakken A.C., Carm K.T., Hoff A.M., Myklebost O., Meza-Zepeda L.A., Lie A.K. (2019). Multifocal Primary Prostate Cancer Exhibits High Degree of Genomic Heterogeneity. Eur. Urol..

[12] Wei L., Wang J., Lampert E., Schlanger S., DePriest A.D., Hu Q., Gomez E.C., Murakam M., Glenn S.T., Conroy J. (2017). Intratumoral and Intertumoral Genomic Heterogeneity of Multifocal Localized Prostate Cancer Impacts Molecular Classifications and Genomic Prognosticators. Eur. Urol..

[13] Gonzalez L., Eiro N., Fernandez-Garcia B., Gonzalez L.O., Dominguez F., Vizoso F.J. (2016). Gene expression profile of normal and cancer-associated fibroblasts according to intratumoral inflammatory cells phenotype from breast cancer tissue. Mol. Carcinog..

[14] Eiro N., Gonzalez L., Martinez-Ordonez A., Fernandez-Garcia B., Gonzalez L.O., Cid S., Dominguez F., Perez-Fernandez R., Vizoso F.J. (2018). Cancer-associated fibroblasts affect breast cancer cell gene expression, invasion and angiogenesis. Cell Oncol..

[15] Macklin M.T. (1954). The genetic basis of human mammary cancer. Proceedings of Second National Cancer Conference.

[16] Lee S.O., Lou W., Johnson C.S., Trump D.L., Gao A.C. (2004). Interleukin-6 protects LNCaP cells from apoptosis induced by androgen deprivation through the Stat3 pathway. Prostate.

[17] Nakashima J., Tachibana M., Horiguchi Y., Oya M., Ohigashi T., Asakura H., Murai M. (2000). Serum interleukin 6 as a prognostic factor in patients with prostate cancer. Clin. Cancer Res..

[18] Gong Y., Chippada-Venkata U.D., Oh W.K. (2014). Roles of matrix metalloproteinases and their natural inhibitors in prostate cancer progression. Cancers.

[19] Vizoso F.J., Gonzalez L.O., Corte M.D., Rodriguez J.C., Vazquez J., Lamelas M.L., Junquera S., Merino A.M., Garcia-Muniz J.L. (2007). Study of matrix metalloproteinases and their inhibitors in breast cancer. Br. J. Cancer.

[20] Gonzalez L.O., Gonzalez-Reyes S., Marin L., Gonzalez L., Gonzalez J.M., Lamelas M.L., Merino A.M., Rodriguez E., Pidal I., del Casar J.M. (2010). Comparative analysis and clinical value of the expression of metalloproteases and their inhibitors by intratumour stromal mononuclear inflammatory cells and those at the invasive front of breast carcinomas. Histopathology.

[21] Eiro N., Fernandez-Garcia B., Vazquez J., Del Casar J.M., Gonzalez L.O., Vizoso F.J. (2015). A phenotype from tumor stroma based on the expression of metalloproteases and their inhibitors, associated with prognosis in breast cancer. Oncoimmunology.

[22] Eiro N., Gonzalez L., Gonzalez L.O., Fernandez-Garcia B., Lamelas M.L., Marin L., Gonzalez-Reyes S., del Casar J.M., Vizoso F.J. (2012). Relationship between the inflammatory molecular profile of breast carcinomas and distant metastasis development. PLoS ONE.

[23] Eiro N., Pidal I., Fernandez-Garcia B., Junquera S., Lamelas M.L., del Casar J.M., Gonzalez L.O., Lopez-Muniz A., Vizoso F.J. (2012). Impact of CD68/(CD3+CD20) ratio at the invasive front of primary tumors on distant metastasis development in breast cancer. PLoS ONE.

[24] Escaff S., Fernandez J.M., Gonzalez L.O., Suarez A., Gonzalez-Reyes S., Gonzalez J.M., Vizoso F.J. (2010). Study of matrix metalloproteinases and their inhibitors in prostate cancer. Br. J. Cancer.

[25] Fernandez-Gomez J., Escaf S., Gonzalez L.O., Suarez A., Gonzalez-Reyes S., Gonzalez J., Miranda O., Vizoso F. (2011). Relationship between metalloprotease expression in tumour and stromal cells and aggressive behaviour in prostate carcinoma: Simultaneous high-throughput study of multiple metalloproteases and their inhibitors using tissue array analysis of radical prostatectomy samples. Scand. J. Urol. Nephrol..

[26] Peruzzi D., Mori F., Conforti A., Lazzaro D., De Rinaldis E., Ciliberto G., La Monica N., Aurisicchio L. (2009). MMP11: A novel target antigen for cancer immunotherapy. Clin. Cancer Res..

[27] Brasse D., Mathelin C., Leroux K., Chenard M.P., Blaise S., Stoll I., Tomasetto C., Rio M.C. (2010). Matrix metalloproteinase 11/stromelysin-3 exerts both activator and repressor functions during the hematogenous metastatic process in mice. Int. J. Cancer.

[28] Boulay A., Masson R., Chenard M.P., El Fahime M., Cassard L., Bellocq J.P., Sautes-Fridman C., Basset P., Rio M.C. (2001). High cancer cell death in syngeneic tumors developed in host mice deficient for the stromelysin-3 matrix metalloproteinase. Cancer Res..

[29] Kanharat N., Tuamsuk P. (2015). Correlation between Microvascular Density and Matrix Metalloproteinase 11 Expression in Prostate Cancer Tissues: A Preliminary Study in Thailand. Asian Pac. J. Cancer Prev..

[30] Ishii K., Takahashi S., Sugimura Y., Watanabe M. (2018). Role of Stromal Paracrine Signals in Proliferative Diseases of the Aging Human Prostate. J. Clin. Med..

[31] Gasinska A., Jaszczynski J., Rychlik U., Luczynska E., Pogodzinski M., Palaczynski M. (2020). Prognostic Significance of Serum PSA Level and Telomerase, VEGF and GLUT-1 Protein Expression for the Biochemical Recurrence in Prostate Cancer Patients after Radical Prostatectomy. Pathol. Oncol. Res..

[32] Verzella D., Fischietti M., Capece D., Vecchiotti D., Del Vecchio F., Cicciarelli G., Mastroiaco V., Tessitore A., Alesse E., Zazzeroni F. (2016). Targeting the NF-kappaB pathway in prostate cancer: A promising therapeutic approach?. Curr. Drug Targets.

[33] Niu Y., Altuwaijri S., Yeh S., Lai K.P., Yu S., Chuang K.H., Huang S.P., Lardy H., Chang C. (2008). Targeting the stromal androgen receptor in primary prostate tumors at earlier stages. Proc. Natl. Acad. Sci. USA.

[34] Vilamaior P.S., Taboga S.R., Carvalho H.F. (2005). Modulation of smooth muscle cell function: Morphological evidence for a contractile to synthetic transition in the rat ventral prostate after castration. Cell Biol. Int..

[35] Yu S., Xia S., Yang D., Wang K., Yeh S., Gao Z., Chang C. (2013). Androgen receptor in human prostate cancer-associated fibroblasts promotes prostate cancer epithelial cell growth and invasion. Med. Oncol..

[36] Thalmann G.N., Rhee H., Sikes R.A., Pathak S., Multani A., Zhau H.E., Marshall F.F., Chung L.W. (2010). Human prostate fibroblasts induce growth and confer castration resistance and metastatic potential in LNCaP Cells. Eur. Urol..

[37] Chen P.-Y., Wei W.-F., Wu H.-Z., Fan L.-S., Wang W. (2021). Cancer-Associated Fibroblast Heterogeneity: A Factor That Cannot Be Ignored in Immune Microenvironment Remodeling. Front. Immunol..

[38] De P., Aske J., Sulaiman R., Dey N. (2022). Bête Noire of Chemotherapy and Targeted Therapy: CAF-Mediated Resistance. Cancers.

[39] Bianchi-Frias D., Basom R., Delrow J.J., Coleman I.M., Dakhova O., Qu X., Fang M., Franco O.E., Ericson N.G., Bielas J.H. (2016). Cells Comprising the Prostate Cancer Microenvironment Lack Recurrent Clonal Somatic Genomic Aberrations. Mol. Cancer Res..

