# Supplementary material for: Gene Expression Profile of Stromal Factors in Cancer-Associated Fibroblasts from Prostate Cancer
Source: Diagnostics (Basel). 2022 Jun 30;12(7):1605. doi: 10.3390/diagnostics12071605 (PMC9325062; doi:10.3390/diagnostics12071605)
Supplement: Supplementary file 1 [file diagnostics-12-01605-s001.zip › diagnostics-1673202-supplementary.pdf]

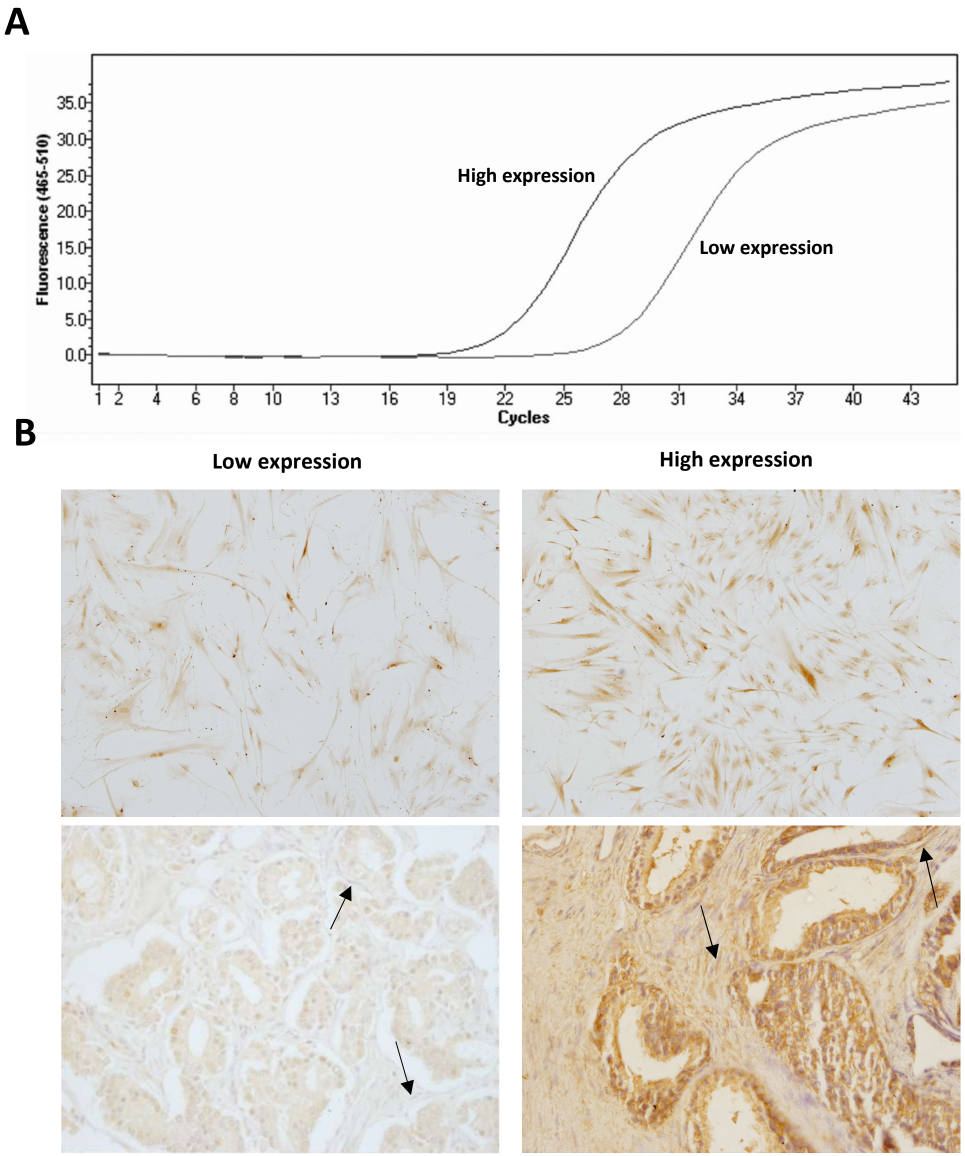

Figure S1: Representative examples of high and low MMP11 expression, both qRT-PCR tran-script level (**A**) and protein (**B**) by CAFs (upper panel) and by prostatectomy cancer tissue (lower panel).
